# Study on Water-Jet-Guided Laser–Ultrasonic Grinding Hybrid Processing Technology for SiC_f_/SiC Composite Materials

**DOI:** 10.3390/ma19112335

**Published:** 2026-06-01

**Authors:** Guanting Yu, Dahai Zhang, Xianjun Pei, Feng Yang

**Affiliations:** 1 School of Mechanical Engineering, Southeast University (SEU), Nanjing 210096, China; 2 School of Mechanical Engineering, Dalian University of Technology, Dalian 116024, China

**Keywords:** SiC_f_, water-jet-guided laser, ultrasonic grinding, composite, efficiency, damage

## Abstract

This study proposes a water-jet guidance–ultrasonic grinding hybrid process for drilling holes in ceramic matrix composites to address issues such as cracking, chipping, thermal damage, and low machining efficiency, thereby achieving high-efficiency, high-quality processing of such components. With final surface quality as the constraint, three methods—water-jet guidance, ultrasonic grinding, and the combined water-jet guidance–ultrasonic grinding—were applied to drill holes in SiC_f_/SiC composite materials. The study investigated how different processes and parameters affect hole surface quality and machining efficiency. Results demonstrate that the hybrid process effectively overcomes defects inherent to single-process grinding (crazing, chipping) and laser processing (heat-affected zones, recast layers), while significantly removing surface oxides generated by water-jet-guided machining. The hole diameter deviation (<0.017 mm), hole wall surface roughness (Sa < 1.837 μm), and processing efficiency (5.25 min/hole) achieved by this composite process all outperformed those of either ultrasonic grinding or water-jet-guided processing alone. This composite process significantly enhances both processing efficiency and hole wall quality, providing a viable solution for high-quality, efficient machining of ceramic matrix composite components.

## 1. Introduction

Silicon carbide fiber-reinforced silicon carbide ceramic matrix (SiC_f_/SiC) composites offer performance advantages such as low density, high hardness, high-temperature resistance, and low thermal expansion coefficient, making them widely used in high-end aerospace hot-end components [1]. The manufacturing process for SiC_f_/SiC composite components primarily involves material preparation, machining, and assembly. Machining focuses on shaping the component’s contours, surfaces, and connection holes. Connection holes represent a critical structural feature of ceramic matrix composites, where machining quality directly impacts component performance and is essential for meeting connection requirements, making it indispensable in the manufacturing process. However, SiC_f_/SiC exhibits characteristics such as high hardness, high brittleness, and anisotropy, making it a typical difficult-to-machine material [2]. When processed using traditional machining techniques, it is highly prone to damage, such as cracks and chipping at the entry and outlet points, which adversely affects the fatigue performance of the component. Therefore, achieving efficient, low-damage machining of connection holes in SiC_f_/SiC composites presents a highly challenging task.

To address the processing bottlenecks of SiC_f_/SiC composites that traditional techniques struggle to overcome, researchers have extensively explored specialized processing technologies [3]. Water-jet-guided laser cutting, a novel processing technique integrating water jets with lasers [4], offers advantages such as uniform material removal, water-cooled processing zones, efficient chip evacuation, and minimal taper. It enables high-quality cutting and small-diameter hole processing with large depth-to-diameter ratios, making it an efficient method for drilling holes in composite parts. Zhang et al. [5] employed water-jet-guided laser technology to process micro-holes in SiC_f_/SiC composites. Results demonstrated smooth hole walls free of slag adhesion post-processing, significantly mitigating thermal ablation issues compared to conventional lasers. Cheng et al. [6] conducted water-jet-guided laser single-point ablation and cutting tests on 3 mm-thick 3D woven SiC_f_/SiC composites. Results demonstrated smooth microhole edges without thermal damage. Although water-jet guided laser technology can avoid the thermal damage issues faced by traditional laser techniques, and the use of an argon atmosphere more effectively suppresses the formation of recast layers, its processing efficiency still needs to be further improved [7]. Therefore, there is an urgent need to explore efficient and low-damage processing techniques for connecting holes in SiC_f_/SiC composite materials using water-jet guided laser processing.

Ultrasonic grinding technology demonstrates significant benefits when machining connection holes in SiC_f_/SiC composite materials, notably reducing drilling forces, minimizing tool wear, and enhancing hole consistency. Extensive research has been conducted by numerous scholars [8,9,10]. Gu et al. [11] investigated the cutting forces and defect formation mechanisms during ultrasonic-assisted drilling of SiC_f_/SiC composites. Results indicated that chipping de-fects dominated on the entry surface, with minor tearing defects observed in localized areas. Compared to conventional drilling, ultrasonic vibration grinding at 5 μm re-duced these defects by approximately 208.5%. Zang et al. [12] investigated the influ-ence of ultrasonic parameters on grinding forces during ultrasonic vibration helical grinding of SiC_f_/SiC composite materials. Experimental results indicate that higher ul-trasonic amplitudes (A = 4 µm and A = 6 µm), lower helical feed rates, and smaller pitches (ap = 0.01 mm/r and ap = 0.02 mm/r) effectively reduce grinding forces. Chen et al. [13] employed ultrasonic vibration-assisted helical grinding technology for micro-pore machining of SiC_f_/SiC composites, observing the morphology and roughness of micro-pores in different orientations. Ultrasonic vibration altered the mechanism of abrasive particle removal from fibers and suppressed crack propagation in the silicon carbide matrix during machining, thereby reducing defects. Experimental results indicate that when the ultrasonic amplitude increased from 0μm to 8μm, the surface roughness decreased from 1.632 μm to 1.172 μm. Lin et al. [14] employed ultrasonic scribing of 2D-SiC_f_/SiC specimens to investigate the material removal mechanism during ultrasonic vibration-assisted grinding of SiC_f_/SiC composites. They observed that conventional scraping resulted in severe fiber delam-ination and detachment, along with greater matrix fracture. Under ultrasonic vibration conditions, fractured fibers shortened, chips became smaller, crack fractures dimin-ished, and overall surface smoothness improved relatively. The above findings indicate that while ultrasonic grinding technology significantly improves machining quality and efficiency compared to conventional grinding, issues such as chipping at the hole outlet and poor dimensional consistency persist when machining a large number of holes or removing substantial material, particularly after tool wear [15].

Researchers have conducted studies on machining connection holes in SiC_f_/SiC composite materials using both waterjet laser and ultrasonic grinding technologies independently, yielding significant conclusions and results. While each technique possesses distinct advantages, its limitations remain apparent when employed individually. When using water-jet-guided method alone to process connection holes in SiC_f_/SiC composites, cracks and chipping frequently occur near the exit due to insufficient material thickness to withstand water erosion, often resulting in costly part scrap. When using ultrasonic grinding alone to process SiC_f_/SiC composite materials, severe tool wear persists due to the material’s high hardness. This prevents ensuring the machining accuracy of high-precision components, necessitating multiple compensation machining passes. This significantly impacts both machining efficiency and machining accuracy.

Research on water-jet guidance–ultrasonic grinding hybrid machining technology remains limited. Wang et al. [16] proposed a laser ablation pretreatment-assisted ultrasonic vibration milling method. After preparing specimens using this process, tensile strength and fatigue performance tests were conducted. Results indicate that this process enhances surface quality, increasing the tensile strength and residual tensile strength of specimens by 9.4% and 13.5%, respectively. Kong et al. [17] conducted comparative experiments on SiC_f_/SiC ceramic matrix composites using conventional grinding and laser-assisted grinding. The study revealed that laser heating induces oxidation reactions in the SiC_f_/SiC ceramic matrix composite, softening the SiC matrix. This reduces grinding forces and alters the material removal mechanism. Compared to conventional grinding, laser-assisted grinding decreased axial force, feed force, and radial force by 43.8%, 40.9%, and 7.8%, respectively. However, the influence patterns of the combined water-jet guided laser and ultrasonic grinding process on surface integrity remain unclear, and the impact of parameter matching on machining performance warrants further investigation. Based on this, this paper innovatively proposes a water-jet guidance–ultrasonic grinding hybrid machining process. This method employs water-jet-guided drilling for efficient bulk material removal, followed by precision grinding to eliminate surface heat-affected zones and microcrack layers. By optimizing the parameter combination of the hybrid process under surface quality constraints, high-quality and efficient hole machining is achieved. Three methods—water-jet guidance, ultrasonic grinding, and water-jet guidance–ultrasonic grinding composite methods to drill holes in SiC_f_/SiC composite materials were applied. The study investigates the influence patterns of different processing methods and parameters on hole entrance/exit morphology, hole diameter, subsurface damage, and processing efficiency. It explores the feasibility of the composite process and optimizes the process parameters for machining SiC_f_/SiC composite materials using the composite process, providing a new pathway for efficient, low-damage processing technology for SiC_f_/SiC composite materials.

## 2. Experimental Setup

### 2.1. Experimental Method

**Figure 1:**
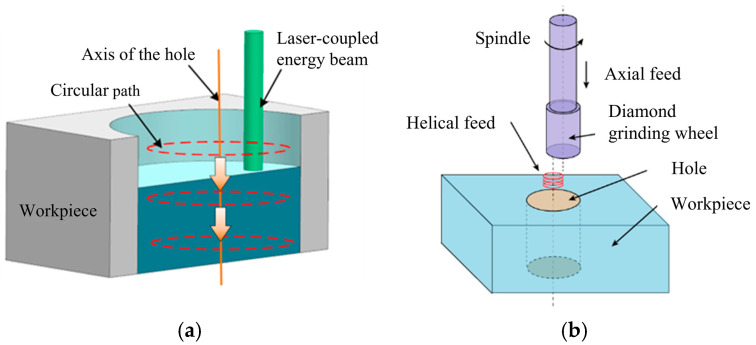
Schematic diagram of ultrasonic grinding and water-jet-guided laser processing. (**a**) Principle of water-jet-guided laser processing; (**b**) principle of ultrasonic grinding.

This study conducted drilling experiments using three distinct process methods: water-jet guided laser drilling, ultrasonic grinding, and a combined water-jet-guidance laser–ultrasonic grinding process. Analyses were performed on the inlet/outlet morphology, bore wall morphology, surface roughness, microstructure, chemical composition, dimensional accuracy, and processing time of holes in ceramic matrix composites. The research investigated the influence of the patterns of these three processing methods on the drilling quality and efficiency of ceramic matrix composites. Water-jet-guided laser processing employed a layered approach using circular paths, while ultrasonic grinding utilized a helical feed method. The processing principles are illustrated in Figure 1.

The test material is a SiC_f_/SiC composite with a specimen thickness of 3.6 mm. The target hole diameter is ⌀3 ± 0.1 mm. No significant burrs or chipping are permitted at the hole inlet/outlet. The wall damage depth must not exceed 10 μm, and the surface roughness Sa must not exceed 2.8 μm.

### 2.2. Water-Jet Guided Laser Drilling

**Figure 2:**
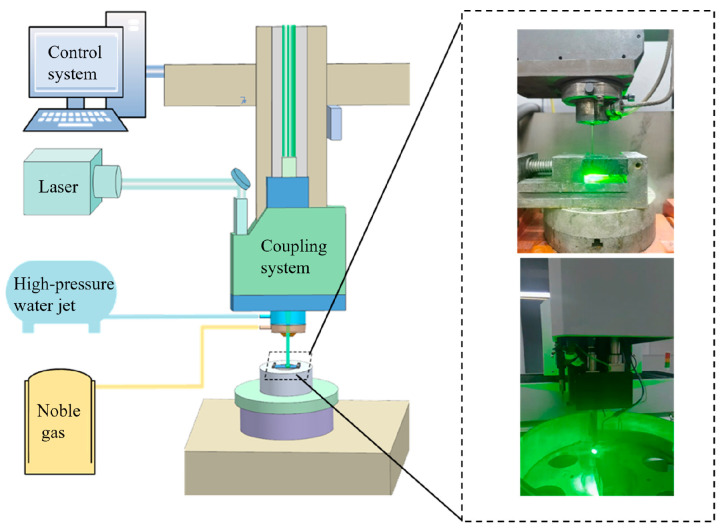
Water-jet-guided laser processing system components.

Water-jet-guided drilling is performed on a water-jet-guided processing machine. The Water-jet-guided processing system comprises a fiber laser (Yinggu Laser, GP 532-100, Suzhou, China), a motion platform, and a water jet generator, as shown in Figure 2. Water-jet guided power exerts the most significant influence on processing quality [18]. This experiment primarily investigates the effects of laser power on the processing quality and efficiency of SiC_f_/SiC composite materials, with a pulse frequency of 10 kHz. The water-jet-guided method achieves an effective processing length of 120–150 mm, eliminating the need for gap adjustments during processing. For the SiC_f_/SiC composite test specimens in this project, preliminary test data guided the selection of laser powers at 20 W, 25 W, 30 W, and 33 W. Since laser head components are prone to ablation damage when power exceeds 33 W, this value was set as the upper limit for testing. The feed rate was 200 mm/min, and the water jet pressure was 20 MPa. Water-jet-guided drilling employed a one-step process to directly achieve the target aperture. Processing parameters are detailed in Table 1. Each test parameter was repeated three times during processing.

### 2.3. Ultrasonic Grinding for Hole Making

**Figure 3:**
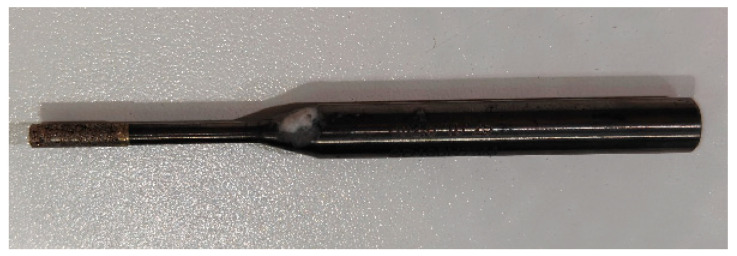
Grinding hole-making tool.

Ultrasonic grinding hole-making tests were conducted on a three-axis grinding machining center. The process involved rough grinding to ⌀2.8 mm followed by finish grinding to ⌀3.0 mm. The ultrasonic-assisted grinding hole-making process parameters are shown in Table 2. Tests were performed by varying feed rates and radial cutting depths. To ensure consistency, each parameter set was repeated three times. The ultrasonic vibration frequency was set at 20 kHz with an amplitude of 5 μm. Hole formation employed a helical grinding method with a pitch of 0.01 mm. The spindle speed was maintained at 6000 r/min. The coolant is pure water with a resistivity of 18.2 MΩ·cm (at 25 °C). The machining tool consisted of a sintered diamond grinding wheel with a diameter of ⌀2.6 mm, a length of 26 mm, and a shank length of 43 mm, as illustrated in Figure 3. After the grinding wheel wears down, the target hole diameter is achieved through tool compensation.

### 2.4. Water-Jet Guided Laser-Ultrasonic Grinding Composite Hole Formation

**Figure 4:**
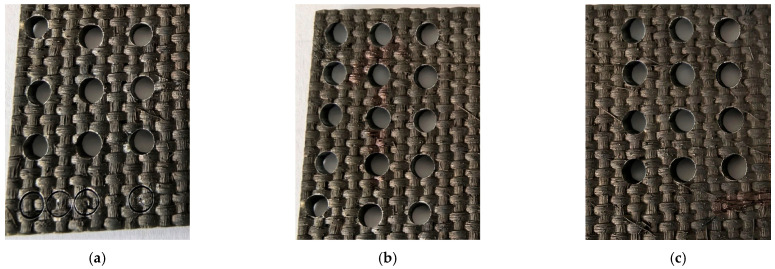
Drilled Sample. (**a**) Water-jet-guided laser; (**b**) ultrasonic grinding; (**c**) water-jet-guided laser-ultrasonic grinding.

The hole-making parameters for the water-jet-guided laser–ultrasonic grinding hybrid process are shown in Table 3. The experiment first employed a water-jet guided laser to drill holes in ceramic matrix composites to ⌀2.8 mm, followed by ultrasonic-assisted grinding to achieve ⌀3.0 mm holes. Water-jet-guided laser processing utilized laser powers of 25 W, 30 W, and 33 W, with a feed rate of 200 mm/min and water jet pressure of 20 MPa. For ultrasonic grinding, feed rates of 50 mm/min and 500 mm/min were selected, with a spindle speed of 6000 r/min, radial depth of cut of 0.1 mm, and pitch of 0.01 mm. Water-jet-guided laser, ultrasonic grinding, and water-jet-guided laser–ultrasonic grinding composite machining of hole samples are shown in Figure 4a–c.

## 3. Processing Quality Analysis

**Figure 5:**
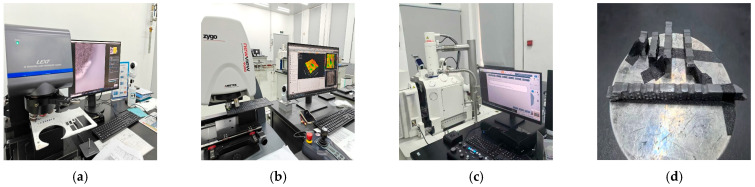
Testing instruments and fixtures used in the experiment. (**a**) Laser confocal microscope; (**b**) 3D surface profiler; (**c**) scanning electron microscope; (**d**) workpiece clamping.

The testing equipment and workpiece clamping method used in this experiment are shown in Figure 5. Following the hole-making test, a high-resolution (0.12 μm) laser confocal microscope (OLYMPUS, OLS5100, OLYMPUS Corporation, Tokyo, Japan) was used to observe the morphology of the hole entrance/exit and hole wall, and to measure the entrance/exit hole diameter. A 3D surface profiler (ZYGO, NewView™ 9000, ZYGO Corporation, Middlefield, CT, USA) was used to measure the surface roughness of the hole wall. A scanning electron microscope (Hitachi, SU3900, Hitachi High-Tech Corporation, Tokyo, Japan) was employed to observe the microstructure and chemical composition of the machined area. Surface composition analysis of the processed region in the SiC_f_/SiC composite material was performed using the energy dispersive spectroscopy (EDS) function of a scanning electron microscope (Hitachi, MC1000, Hitachi High-Tech Corporation, Tokyo, Japan). The EDS analysis was conducted at an operating voltage of 20 kV to ensure effective excitation of the target elements.

To ensure clear, high-quality imaging of specimens under the scanning electron microscope (SEM), samples undergo grinding, polishing, and gold sputtering. The primary steps for gold sputtering are as follows: First, the clean and dry specimen is secured to the sample stage. It is then placed in the sputtering chamber and evacuated to high vacuum. Through ion sputtering, a gold–palladium alloy target is evaporated, uniformly depositing an extremely thin (approximately 5–6 nm) metallic film onto the specimen surface. Finally, the chamber pressure is released, and the specimen is removed, presenting a uniform light golden color. This process effectively neutralizes specimen charge, significantly enhancing imaging clarity and quality.

### 3.1. Analysis of Surface Quality in Water-Jet Guided Laser Drilling

#### 3.1.1. Morphology of the Hole Inlet and Outlet

**Figure 6:**
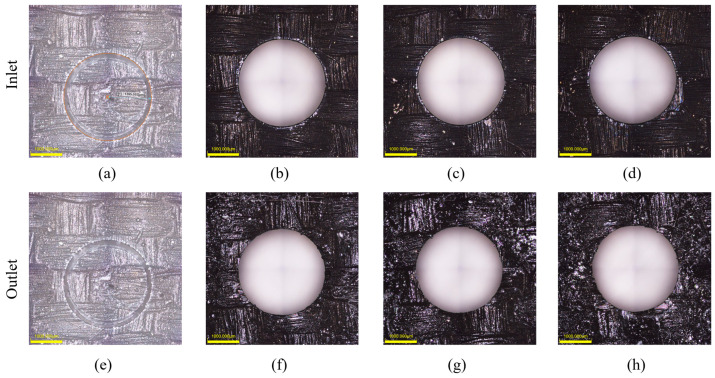
Inlet and outlet morphology of water-jet-guided laser drilling at different power levels. (**a**) 20 W; (**b**) 25 W; (**c**) 30 W; (**d**) 33 W; (**e**) 20 W; (**f**) 25 W; (**g**) 30 W; (**h**) 33 W.

The inlet processing quality at 20 W, 25 W, 30 W, and 33 W power levels is shown in Figure 6a, b, c and d, respectively, while the outlet processing quality is depicted in Figure 6e, f, g and h, respectively. At 20 W, direct processing is not feasible. As laser power increases, processability improves. When laser power reaches 30 W, the surface quality of the inlet and outlet orifices is enhanced. At this point, the heat input from the laser rapidly melts the material, while the high-pressure water jet simultaneously and thoroughly flushes away the molten material, preventing secondary thermal effects. This is facilitated by the stable total internal reflection of the laser within the water jet during water-jet guided-laser processing, ensuring uniform energy distribution. The continuous water flow effectively removes heat from the hole walls, suppressing material overburning and thermal diffusion [19]. When the laser power increased from 30 W to 33 W, the surface quality of the holes at the inlet and outlet deteriorated. This was due to the high laser power causing both ablation and intense vaporization of the material. The vaporization produced high-pressure vapor bubbles that impacted the hole walls, resulting in a rough surface [20].

#### 3.1.2. Hole Wall Morphology

**Figure 7:**
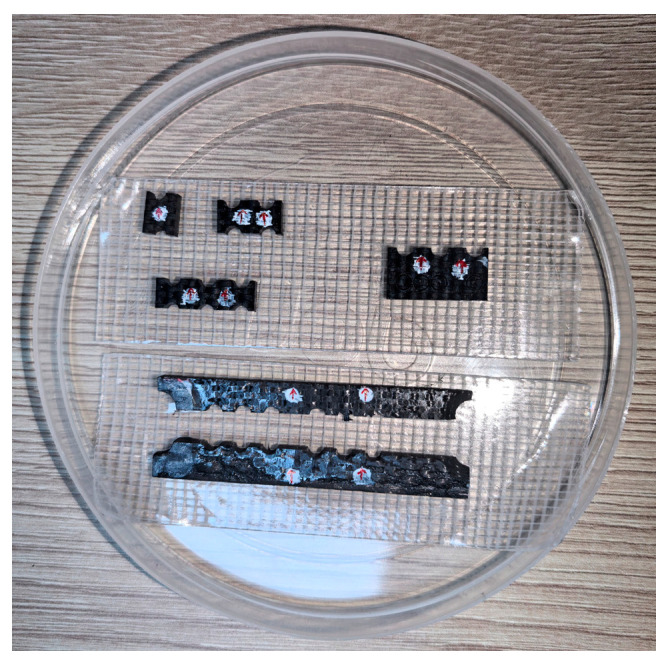
Ceramic matrix composite hole wall specimen.

Pore wall observation samples were obtained through grinding methods, as shown in Figure 7. Laser confocal microscopy combined with image stitching was employed to examine the surface morphology of the pore walls.

**Figure 8:**
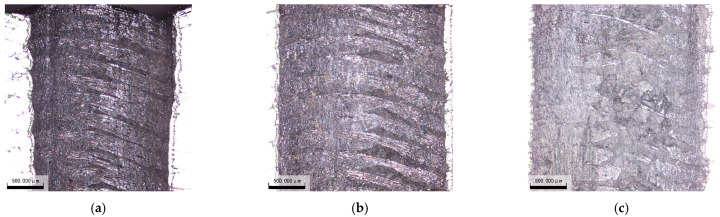
Wall morphology of holes formed by water-jet-guided laser power at different levels. (**a**) 25 W; (**b**) 30 W; (**c**) 33 W.

The surface topography of holes fabricated using 25 W, 30 W, and 33 W laser powers is shown in Figure 8a, b and c, respectively. At high laser powers, micro-flaking and other material defects are more likely to occur on the hole walls. This is because increased water-jet-guided power promotes oxidation and ablation on the processed surface. Simultaneously, the interface between the fiber and substrate is thermally sensitive; high heat causes oxidation or ablation, weakening the bond strength [21]. Under water flow, the molten material and debris are then eroded away.

#### 3.1.3. Surface Roughness

A 3D surface profiler was employed to measure the roughness of the hole walls. Considering the anisotropic characteristics of composite materials, the three-dimensional surface roughness parameter Sa was used to evaluate hole quality. The assessment area size and filtering process adhered to the ISO 25178-2 standard [22]. To ensure result validity, measurements were taken at three distinct positions for each hole, with the average value recorded.

**Figure 9:**
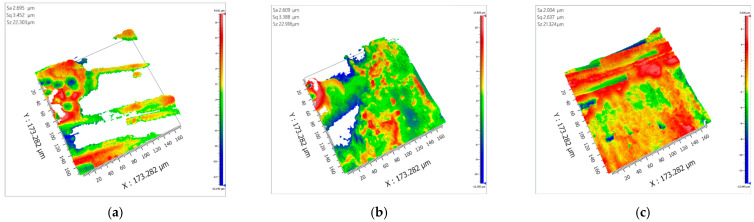
Surface roughness of hole walls produced by water-jet-guided laser drilling at different power levels. (**a**) 25 W; (**b**) 30 W; (**c**) 33 W.

Experimental results indicate that when the water-jet-guided power is 25 W, 30 W, and 33 W, respectively, the average hole wall roughness values are Sa 2.015 μm, Sa 2.606 μm, and Sa 2.705 μm, as shown in Table 4. Measurement results are illustrated in Figure 9. This indicates that within a certain power range, as laser power increases, the ablation effect on the material intensifies, leading to faster material removal and higher surface roughness Sa values.

#### 3.1.4. Microstructure

**Figure 10:**
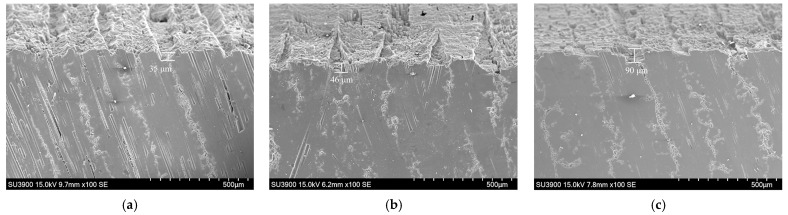
Cross-sectional morphology of hole walls formed by water-jet-guided laser drilling at different power levels. (**a**) 25 W; (**b**) 30 W; (**c**) 33 W.

Ceramic matrix composite test specimens were ground, polished, and gold-sputtered to enhance imaging quality. Scanning electron microscopy was employed to measure and analyze the processing damage depth. Figure 10 shows the cross-sectional profiles of holes produced by a water-jet-guided process at different powers. Processing damage primarily occurred at fiber intersections, manifesting as small-scale wedge-shaped pits. The depth of these pits corresponds to the depth of the processed damage layer. Observing the three locations with the greatest damage depth on the specimens: at 25 W power, the processing damage depth ranged from approximately 30 to 35 μm; at 30 W power, the processing damage depth ranged from approximately 40 to 46 μm; and at 33 W power, the processing damage depth ranged from approximately 80 to 90 μm. The experimental results indicate that the damage depth increases with increasing power. This is because, as the laser power increases, the single-pulse energy increases, significantly enhancing the ablation depth and volumetric removal rate, thereby creating larger pits on the processed surface [23]. Therefore, by controlling the water-jet-guided power, the processing damage depth of the hole wall can be controlled.

#### 3.1.5. Chemical Composition Analysis

**Figure 11:**
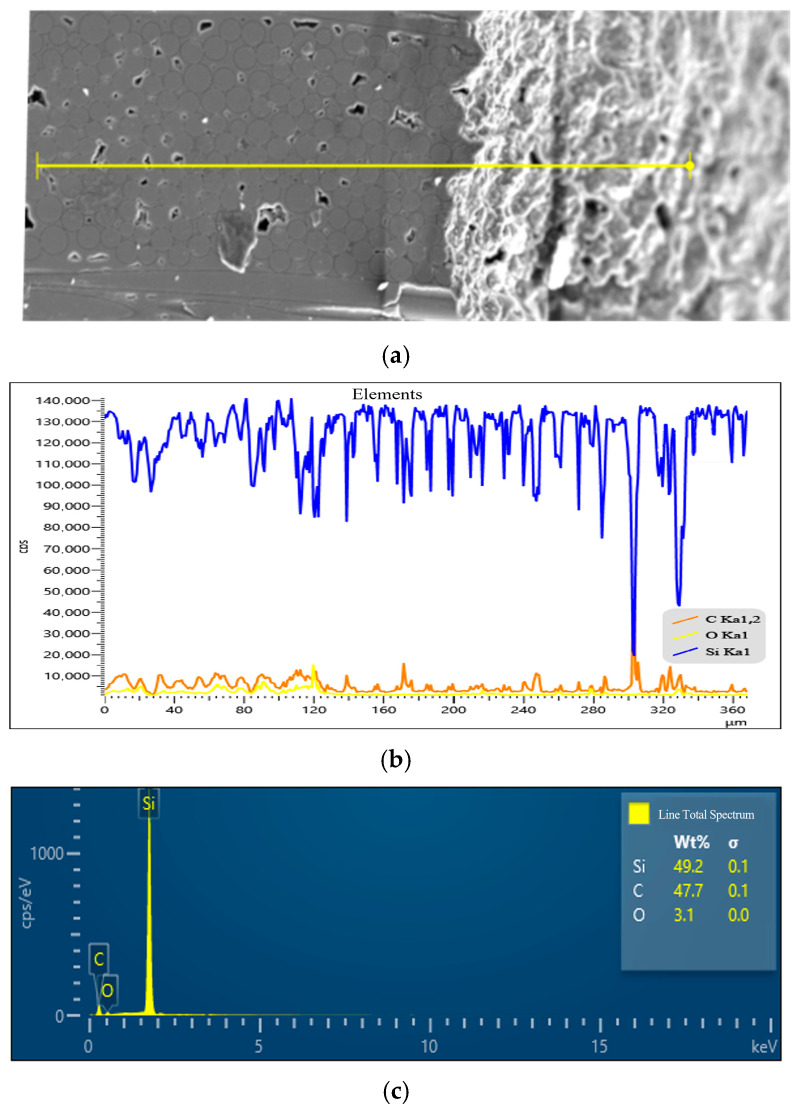
Water-jet guided laser drilling component analysis (25 W). (**a**) Observation path; (**b**) component distribution; (**c**) energy dispersive.

Surface composition analysis of the machined area was performed using the energy dispersive spectroscopy (EDS) function of the scanning electron microscope. The composition analysis of the water-jet-guided laser-drilled hole at 25 W power is shown in Figure 11. The post-processing surface composition consists of C, Si, and O, with mass percentages of 47.7%, 49.2%, and 3.1%, respectively. Along the observation path from the hole wall to the cross-section of the substrate, the C and O content at the hole wall position was higher than that at the substrate position, as shown in Figure 11b. This indicates that oxidation occurred on the wall of the water-jet-guided laser-drilled hole.

**Figure 12:**
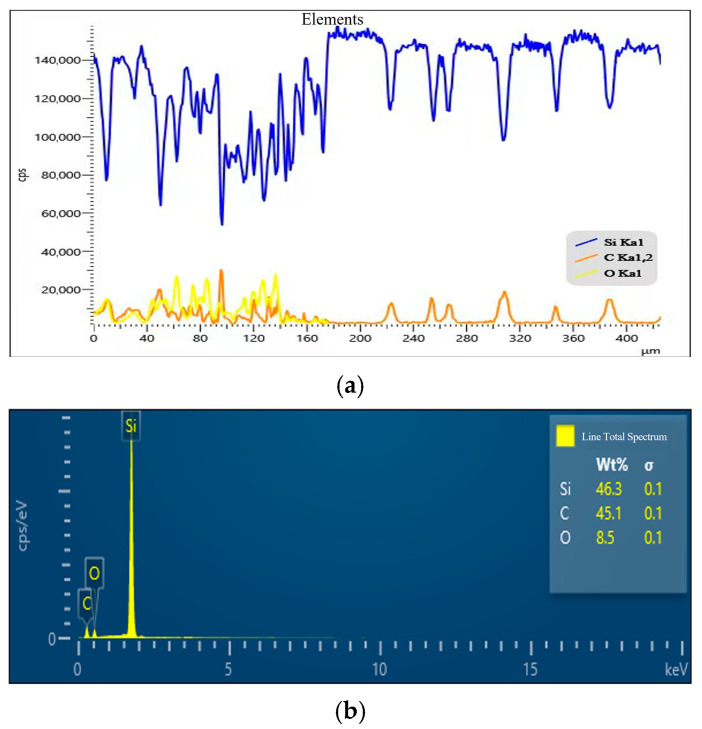
Water-jet-guided laser drilling component analysis (33 W). (**a**) Elementary distribution; (**b**) energy dispersive.

The composition analysis of water-jet-guided laser drilling using 33 W power is shown in Figure 12. The processed surface components are C, Si, and O, with mass percentages of 45.1%, 46.3%, and 8.5%, respectively. Along the observation path from the hole wall to the cross-section of the substrate, the C and O contents at the hole wall position exceed those at the substrate position, as shown in Figure 12a. When the laser power was increased from 25 W to 33 W, the O mass percentage rose from 3.1% to 8.5%, as shown in Figure 12b. This indicates that as the water-jet-guided power increases, the ablation temperature rises, leading to more severe oxidation of the hole walls. The increase in oxidation severity follows the same trend as the increase in microstructural damage depth.

#### 3.1.6. Hole Diameters Deviation

**Figure 13:**
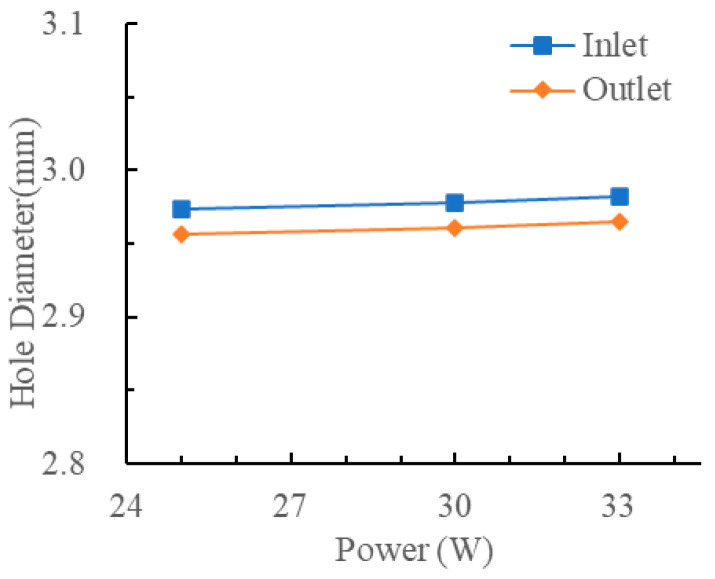
Hole diameter of water-jet-guided laser drilling at different power levels.

Using a laser confocal microscope to measure the hole sizes at the inlet and outlet surfaces, the statistical data for water-jet-guided drilling holes under three laser power levels are shown in Table 5. The average inlet hole diameters were 2.974 mm, 2.978 mm, and 2.983 mm, respectively, while the average outlet hole diameters were 2.956 mm, 2.961 mm, and 2.965 mm, respectively. Experimental data indicate that hole size increases with power, as shown in Figure 13. The slightly larger inlet hole diameters compared to the outlet are attributed to longer laser ablation time at the inlet, resulting in an increased hole size. The maximum difference in diameter between the inlet and outlet at each power level represents the hole diameter deviation. As shown in Table 5, the hole diameter deviation for water-jet-guided drilling is ≤0.04 mm.

### 3.2. Analysis of Surface Quality in Ultrasonic-Assisted Grinding Hole Formation

#### 3.2.1. Hole Inlet and Outlet Morphology

**Figure 14:**
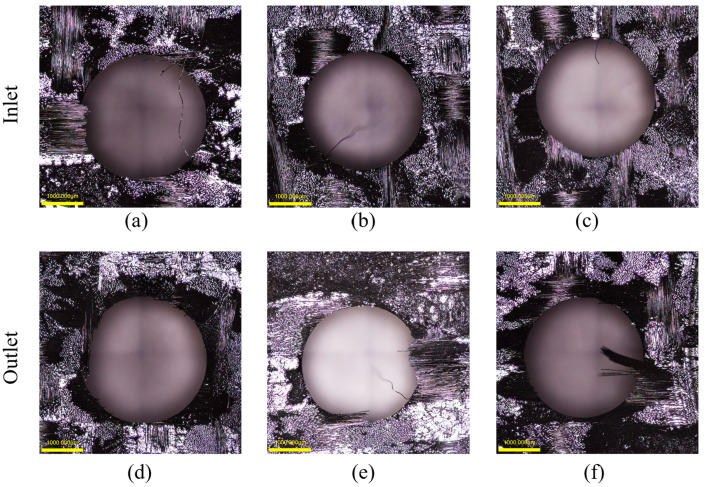
Inlet and outlet morphology of ultrasonic grinding holes at different feed rates. (**a**) F = 50 mm/min; (**b**) F = 200 mm/min; (**c**) F = 500 mm/min; (**d**) F = 50 mm/min; (**e**) F = 200 mm/min; (**f**) F = 500 mm/min.

The morphology of the hole inlet and outlet under ultrasonic-assisted grinding is shown in Figure 14. The inlets exhibit no significant burrs, meeting process requirements. However, as the feed rate increases, burring and chipping on the outlet hole walls intensify. This occurs because the increased feed rate increases the volume of material removed per unit time by each abrasive grain. This significantly increases the thickness of the undeformed chip, causing the instantaneous force acting on a single fiber to exceed its interfacial bond strength with the substrate. However, this force does not yet reach the fiber’s own fracture strength. Due to the lack of support at the hole outlet, when a transverse crack propagating from the interior reaches the free surface, it instantly causes the outlet layer material to peel off, resulting in burrs and chipping. At a second grinding feed rate of 500 mm/min, outlet-quality burrs and chipping persisted. Recommended machining parameters: first rough grinding: 500 mm/min, second finish grinding: 200 mm/min.

#### 3.2.2. Hole Wall Morphology

**Figure 15:**
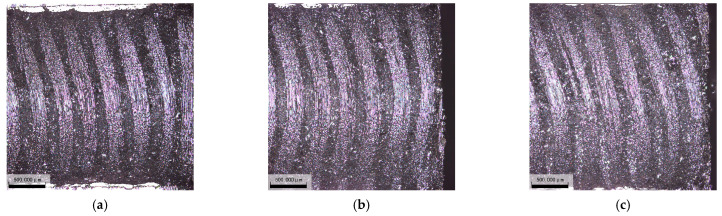
Wall topography of holes formed by grinding at different feed rates. (**a**) F = 50 mm/min; (**b**) F = 200 mm/min; (**c**) F = 500 mm/min.

The surface topography of ultrasonic-assisted grinding holes at different feed rates is shown in Figure 15a, b, and c, respectively. The woven fiber pattern of the ceramic matrix composite is visible in the figures. This indicates that each fiber layer is completely severed during grinding, resulting in a fully formed hole surface. The surface quality of holes produced at a feed rate of 50 mm/min is superior to that at 500 mm/min. This is because at lower feed rates, the material removal rate decreases, leading to a significant increase in the number of effective ultrasonic impacts per unit area. This enhances the ultrasonic processing effect, resulting in improved surface quality of the hole walls.

#### 3.2.3. Surface Roughness

**Figure 16:**
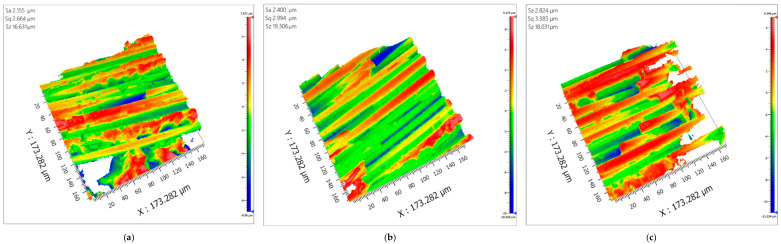
Surface roughness of ultrasonic grinding. (**a**) F = 50 mm/min; (**b**) F = 200 mm/min; (**c**) F = 500 mm/min.

The surface roughness of the hole walls was measured using a 3D surface profiler, with three different positions measured for each hole. The average surface roughness values of the hole walls produced by ultrasonic-assisted grinding at different feed rates were Sa 2.154 μm, Sa 2.417 μm, and Sa 2.867 μm, respectively, as shown in Table 6. An example of the measurement results is illustrated in Figure 16. The figure reveals grinding marks on the bore wall surface. These marks result from abrasive particles compressing and cutting into the workpiece material during grinding, causing scratching, and plowing actions that create fine groove patterns on the material surface. At a feed rate of 500 mm/min, the surface roughness of the ultrasonic grinding process (Sa = 2.824 μm) was slightly higher than the target value (Sa = 2.8 μm).

#### 3.2.4. Microstructure

**Figure 17:**
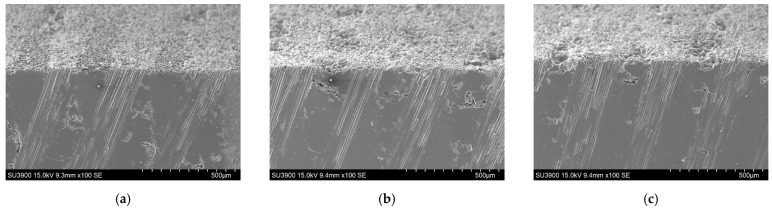
Inlet and outlet morphology of holes produced by helical fine grinding at different feed rates. (**a**) F = 50 mm/min; (**b**) F = 200 mm/min; (**c**) F = 500 mm/min.

The surface topography of ultrasonic-assisted grinding holes at different feed rates is shown in Figure 17a, b and c, respectively. Processing damage primarily occurs at fiber intersections, manifesting as small-scale wedge-shaped pits with damage depths ranging from 5 μm to 10 μm. As the feed rate decreases, the damage depth becomes smaller. This is because under ultrasonic action, material is removed by a single or a few impacts of the abrasive particles, leaving insufficient time for cracks to propagate. Simultaneously, reducing the feed rate enhances the ultrasonic “hammering” effect, shifting the material removal mechanism toward a hybrid mode combining “brittle fracture” and “micro-plastic flow”.

#### 3.2.5. Chemical Composition Analysis

**Figure 18:**
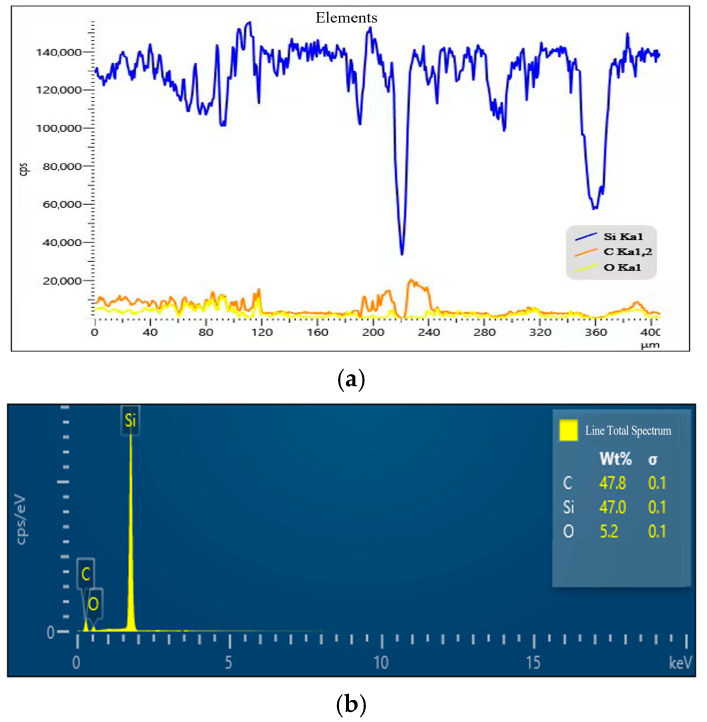
Ultrasonic grinding hole formation component analysis (feed rate 200 mm/min). (**a**) Element distribution; (**b**) energy dispersive.

The compositional analysis of the hole produced by ultrasonic grinding at a feed rate of 200 mm/min is shown in Figure 18. The surface composition after ultrasonic grinding consists of C, Si, and O, with mass percentages of 47.8%, 47.0%, and 5.2%, respectively. Along the observation path from the hole wall surface to the cross-section of the substrate, no significant variation in material composition was observed at the corresponding positions of the hole wall and substrate. This indicates that the ultrasonic grinding process does not affect the composition of the ceramic matrix composite during hole formation.

**Figure 19:**
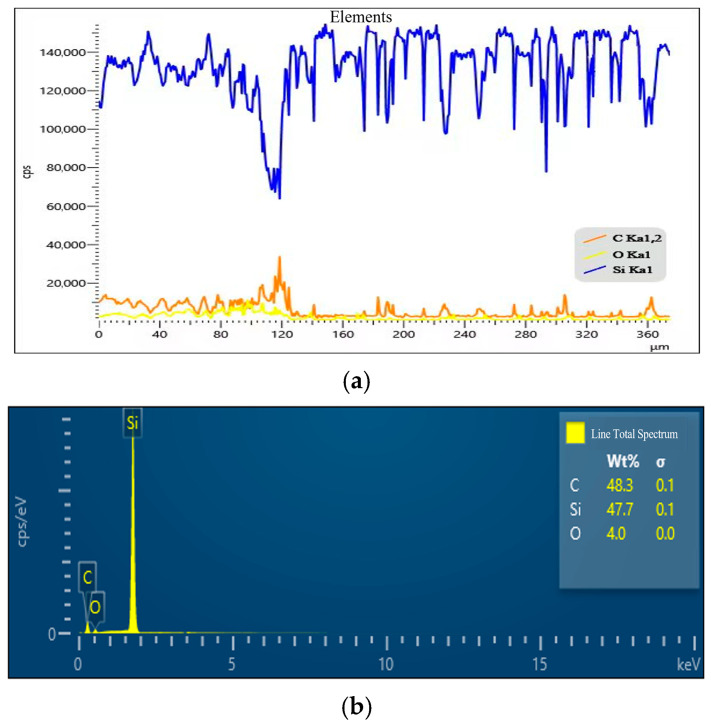
Ultrasonic grinding hole formation component analysis (feed rate 500 mm/min). (**a**) Element distribution; (**b**) energy dispersive.

The compositional analysis of the hole produced by ultrasonic grinding at a feed rate of 500 mm/min is shown in Figure 19. The surface composition after ultrasonic grinding consists of C, Si, and O, with mass percentages of 48.3%, 47.7%, and 4%, respectively. Along the observation path from the hole wall surface to the cross-section of the substrate, the C and O content at the hole wall position was slightly higher than that at the substrate position. This indicates that at high feed rates, as grinding temperature increases, a small amount of oxidation occurs at the hole wall. The damage depth in ultrasonic-assisted grinding hole formation is less than that in water-jet-guided processing, which is attributed to the lower oxidation effect on the hole wall during ultrasonic-assisted grinding.

#### 3.2.6. Hole Diameters Deviation

**Figure 20:**
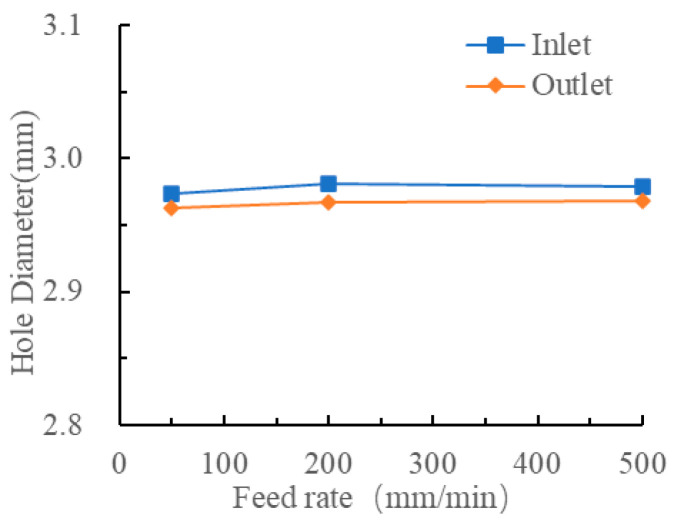
Hole diameter in spiral rough-ground holes at different feed rates.

Laser confocal microscopy was employed to measure the inlet and outlet hole dimensions. The statistical data for the apertures at three feed rates are presented in Table 7. The average inlet hole diameters were 2.974 mm, 2.981 mm, and 2.979 mm, respectively, while the average outlet hole diameters were 2.963 mm, 2.967 mm, and 2.969 mm, respectively. As shown in Figure 20, the average inlet hole diameters slightly exceed the outlet hole diameters. Both hole diameter values fall below the theoretical hole diameters of 3 mm, indicating that during machining, the smaller tool diameter causes a certain degree of “tool deflection” under the tangential grinding force. Additionally, under sustained normal grinding force, material at the exit point is prone to bending or tearing, leading to burrs and edge deformation. Therefore, appropriately reducing the feed rate lowers the average normal grinding force, diminishing the sustained pressure on the exit material. This helps maintain its rigidity, reduces edge burrs caused by tool deflection and elastic recovery, and ultimately achieves a smoother hole exit quality. The maximum difference in diameter between the hole’s entry and exit points at different feed rates constitutes the hole diameter deviation. As shown in Table 7, the hole diameter deviation for ultrasonic grinding holes is ≤0.05 mm.

### 3.3. Analysis of Surface Quality in Water-Jet Guided Laser-Ultrasonic Composite Hole Formation

#### 3.3.1. Inlet and Outlet Morphology

**Figure 21:**
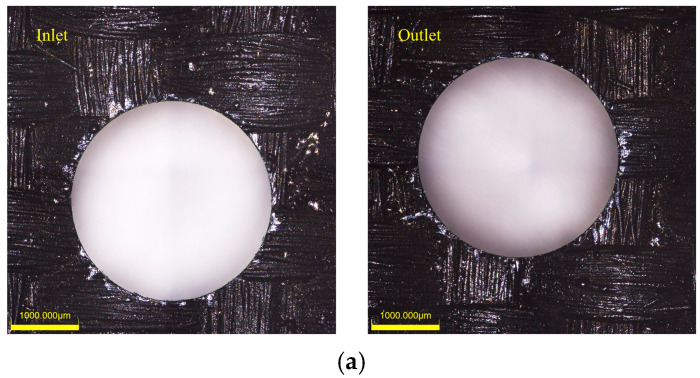

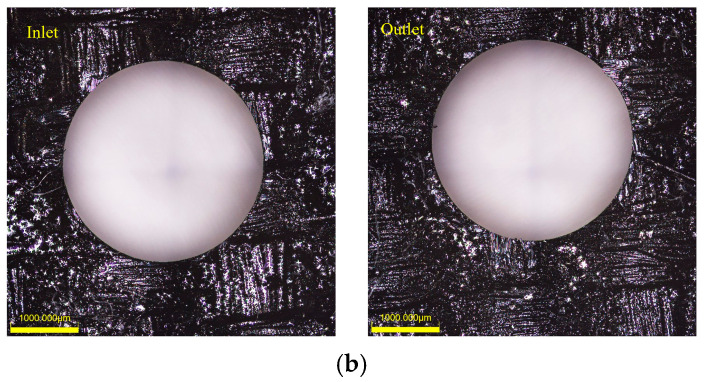
Inlet and outlet morphology of holes produced by water-jet-guided laser–ultrasonic grinding composite process. (**a**) Laser power: 33 W, grinding feed rate: 50 mm/min; (**b**) laser power: 33 W, grinding feed rate: 500 mm/min.

To enhance machining efficiency, a laser power of 33 W was selected for initial hole processing. First, a water-jet-guided drilling was used to create an initial hole with a diameter of φ2.8 mm. Subsequently, grinding processes with feed rates of 50 mm/min and 500 mm/min were employed to finish the hole to φ3.0 mm. Observation using a laser confocal microscope revealed the hole entrance and exit morphologies as shown in Figure 21a,b. The hole walls at the entrance and exit were smooth, with no chipping or other machining damage observed. The hole quality at the entrance and exit produced by the composite grinding process was superior to that achieved by either ultrasonic grinding or water-jet guided drilling alone. The water-jet-guided drilling first achieved efficient material removal, followed by ultrasonic grinding for precision finishing of the hole walls, significantly enhancing the machining quality.

#### 3.3.2. Hole Wall Morphology

**Figure 22:**
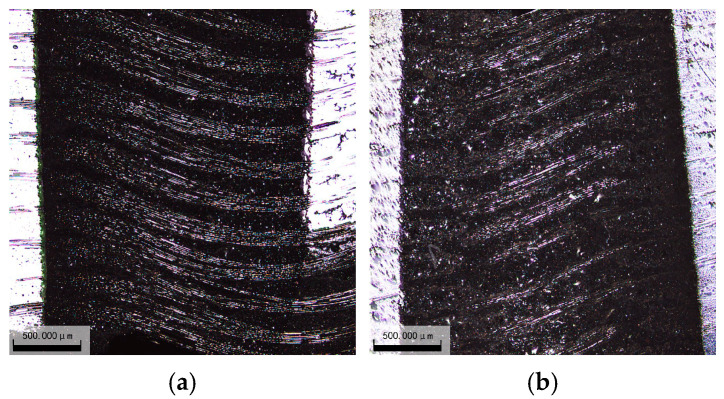
Wall topography of holes processed by water-jet-guided laser-ultrasonic grinding composite process at different feed rates. (**a**) 50 mm/min; (**b**) 500 mm/min.

Laser confocal microscopy was employed to observe the hole wall morphology, as shown in Figure 22. At lower feed rates, the hole wall morphology exhibited uniform micro-cutting marks from abrasive particles, with clean fiber breaks and smooth substrate removal, free from chipping or burrs. As the feed rate increased, high quality was maintained. At the optical microscope scale, no significant difference in machining quality was observed between the two processes. This is attributed to the precise removal of stock material achieved by ultrasonic grinding. To enhance machining speed while maintaining quality, composite process parameters should prioritize higher power and feed rates: 33 W for water-jet-guided processing and 500 mm/min for ultrasonic-assisted grinding.

#### 3.3.3. Surface Roughness

**Figure 23:**
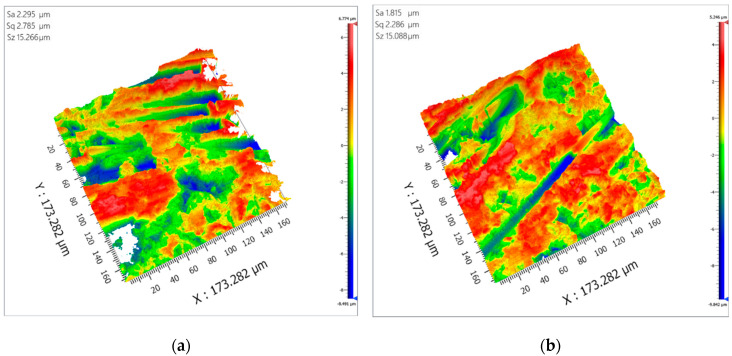
Surface roughness of holes produced by water-jet-guided laser–ultrasonic grinding composite process. (**a**) 33 W, F = 50 mm/min; (**b**) 33 W, F = 500 mm/min.

At two feed rates, the average surface roughness values of the hole walls were Sa 2.303 μm and Sa 1.837 μm, respectively, as shown in Table 8. Measurement results are illustrated in Figure 23. The figure reveals grinding marks on the bore wall surface. This indicates that after water-jet-guided processing, the workpiece material underwent grinding to alter the surface profile formed by laser processing. Both composite processes yielded favorable surface roughness values. The lowest surface roughness value of Sa 1.815 μm was achieved at a water-jet-guided power of 33 W and a grinding feed rate of 500 mm/min. This demonstrates that after oxidation and ablation induced by the laser, the material becomes more amenable to grinding removal. The resulting surface quality surpasses that achieved by either water-jet-guided processing or ultrasonic grinding alone.

#### 3.3.4. Microstructure

**Figure 24:**
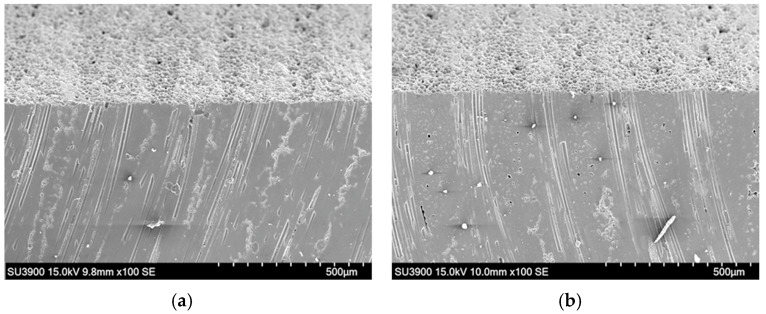
Wall topography of holes produced by water-jet-guided laser–ultrasonic composite grinding at different feed rates. (**a**) 33 W, F = 50 mm/min; (**b**) 33 W, F = 500 mm/min.

The wall morphology of holes produced by water-jet-guided laser–ultrasonic grinding at different feed rates is shown in Figure 24. When laser power was set to 33 W and feed rates of 50 mm/min and 500 mm/min were applied, both yielded excellent machining quality with no visible surface damage. This occurs because laser energy ablates the surface layer, significantly reducing its yield strength and fracture toughness. Ultrasonic grinding efficiently removes material with lower impact forces and micro-fracturing mechanisms, simultaneously and thoroughly grinding away the extremely thin heat-affected zone and trace molten material generated by the laser. Consequently, the hybrid process enables high-quality, low-damage machining of holes in ceramic matrix composites. Thus, the hybrid process offers the advantage of superior machining quality. Considering machining efficiency, a feed rate of 500 mm/min is prioritized for processing.

#### 3.3.5. Chemical Composition Analysis

**Figure 25:**
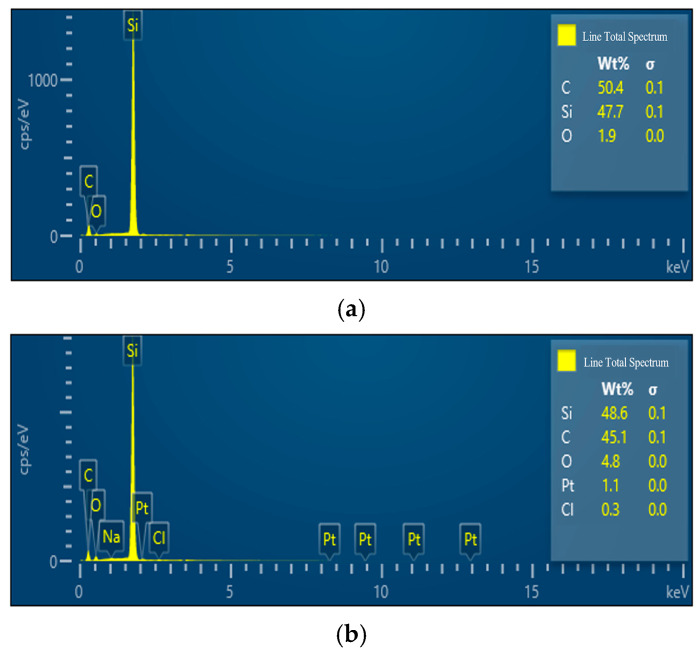
Water-jet-guided laser-ultrasonic grinding composite hole-making component analysis. (**a**) 33 W, F = 50 mm/min; (**b**) 33 W, F = 500 mm/min.

Surface composition analysis of the processed area was performed using the energy dispersive spectroscopy (EDS) function of the scanning electron microscope, as shown in Figure 25a,b. The post-processing surface composition comprised C, Si, and O. At a feed rate of 50 mm/min, their mass percentages were 50.4%, 47.7%, and 1.9%, respectively. At 50 mm/min, the mass percentages were 45.1%, 48.6%, and 4.8%. Additionally, elements such as Pt were detected, attributed to the spray gold process. Along the observation path, the mass percentages of C and O showed no significant variation, indicating that the composite grinding process effectively removes the oxide layer from the bore wall, resulting in a machined surface composition close to that of the base material.

#### 3.3.6. Hole Diameters Deviation

**Figure 26:**
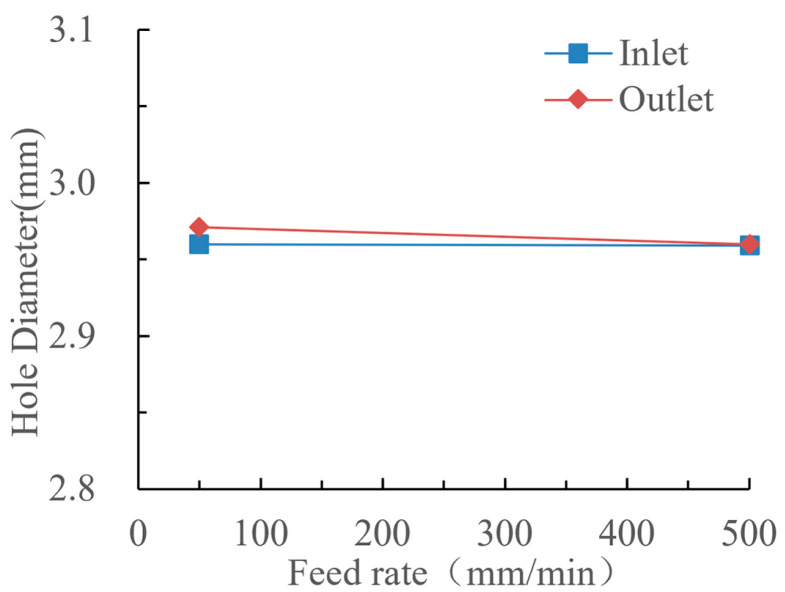
Average hole diameter obtained through water-jet-guided laser–ultrasonic grinding hole-making process.

The hole diameters produced by the water-jet guidance–ultrasonic grinding composite process are shown in Table 9. At a laser power of 33 W and two feed rates, the average inlet diameters were 2.960 mm and 2.959 mm, while the average outlet diameters were 2.971 mm and 2.960 mm. As shown in Figure 26, the composite process produces holes with excellent consistency in inlet and outlet diameters, with a diameter deviation of ≤0.017 mm. This is because after removing the large stock with water-jet-guided processing, the oxide layer formed on the hole wall exhibits low hardness, high brittleness, and easy detachment. Its hardness is only 1/3 to 1/2 that of the SiC substrate. Furthermore, as the power of water-jet-guided processing increases, the depth of the oxide layer on the hole wall also increases [24], making it easier to achieve high dimensional accuracy through ultrasonic grinding. The test results demonstrate the advantage of the composite processing technique in enhancing hole-making precision.

### 3.4. Comprehensive Analysis

#### 3.4.1. Surface Roughness

The wall roughness of holes produced by water-jet-guided drilling is ≤Sa 2.705 μm, while that of ultrasonic grinding is ≤Sa 2.417 μm. Holes fabricated using the water-jet guidance–ultrasonic grinding hybrid process exhibit wall roughness ≤ Sa1.837 μm. All three processing methods achieve hole wall surface roughness ≤ Sa 2.8 μm. The surface roughness of holes produced by the water-jet guidance–ultrasonic grinding composite process outperforms both ultrasonic grinding and water-jet-guided drilling. This superiority stems from the composite process’s ultrasonic grinding step, which performs precision grinding on the blank stock, enabling meticulous refinement of the hole wall material.

#### 3.4.2. Hole Diameter Deviation

The hole diameter machining deviation of water-jet-guided laser drilling is <0.04 mm, while that of ultrasonic grinding is <0.05 mm. The hole diameter machining deviation achieved by the water-jet-guided laser-ultrasonic grinding hybrid process is <0.017 mm, with inlet and outlet dimensions closer to the theoretical hole diameter, outperforming both ultrasonic grinding and water-jet-guided laser drilling. Therefore, the water-jet- guided laser-ultrasonic grinding composite process achieves superior machining quality compared to either ultrasonic grinding or water-jet-guided laser drilling alone.

#### 3.4.3. Surface Quality of Machined Holes

The damage layer depth for water-jet-guided laser drilling is ≤90 μm, while that for ultrasonic drilling is ≤10 μm. The bore walls produced by water-jet-guided laser-composite grinding exhibit no damage. Therefore, the composite processing technique combines the high-efficiency machining of water-jet-guided laser with the low-damage advantage of ultrasonic grinding, enabling near-zero-damage hole formation.

## 4. Processing Efficiency Analysis

Surface ablation and oxidation occur on laser-drilled holes. These can be mitigated by applying ultrasonic-assisted grinding to the machined surfaces for finishing, thereby improving surface quality and enhancing dimensional accuracy. An analysis of the machining efficiency for three process methods was conducted to identify the overall optimal process solution. In this study, the processing time refers to the actual machining time for a single hole, excluding setup time and auxiliary time. Additionally, due to the low hardness of the oxide layer formed on surfaces after water-jet-guided processing, the grinding wheel wear in the composite process is significantly lower than that in the ultrasonic grinding process. However, the composite process increases the number of process steps and setups, which also affects the total manufacturing time. In actual part machining, analysis should be conducted based on specific circumstances.

### 4.1. Analysis of Water-Jet Guided Laser Drilling Efficiency

The processing time statistics for water-jet-guided laser drilling at different power levels are shown in Table 10. As the power increased from 25 W to 33 W, the processing time decreased from 12 min to 3 min. This is because the primary mechanism of material removal is stable melting. Higher power enables the material to reach an ablation state more rapidly, allowing it to be eroded and carried away by the water jet in a more continuous and accelerated manner, thereby reducing the processing time.

### 4.2. Analysis of Hole-Making Efficiency in Ultrasonic-Assisted Grinding

Statistics on ultrasonic-assisted grinding spiral hole drilling times are shown in Table 11. The total machining time for the grinding process is the sum of the two grinding steps. As the feed rate increased from 50 mm/min to 500 mm/min, machining efficiency improved, reducing the total processing time from 22.5 min to 2.25 min. For this experiment, the optimal ultrasonic helical hole-making scheme to meet process objectives is as follows: First, rough grind to ⌀2.8 mm at a feed rate of 500 mm/min, with a processing time of 1.12 min. Then, finish grinding to ⌀3 mm at 200 mm/min, with a processing time of 5.6 min. The total processing time is 6.72 min.

### 4.3. Efficiency Analysis of Water-Jet-Guided Laser–Ultrasonic Grinding Composite Hole-Making Process

The optimal process scheme for water-jet-guided laser–ultrasonic grinding composite drilling is as follows: First, rough-machine to ⌀2.8 mm using 33 W laser power and a feed rate of 200 mm/min, with a processing time of 3 min. Then, perform ultrasonic helical drilling at 500 mm/min to achieve ⌀3.0 mm, with a processing time of 2.25 min. The total composite process time is 5.25 min, as shown in Table 12.

In conclusion, Table 13 presents a comprehensive comparison of the three machining methods in terms of surface roughness, hole diameter deviation, surface quality, and machining efficiency. The water-jet guidance–ultrasonic grinding composite hole-making process effectively enhances the wall quality, diameter deviation, and machining efficiency of holes in SiC_f_/SiC composite parts.

## 5. Conclusions

This study employed three distinct machining methods to create holes in SiC_f_/SiC composite materials, investigating the influence of different processes and parameters on hole machining quality and efficiency. Through optimized parameter matching experiments for water-jet guidance and ultrasonic grinding processes, efficient and low-damage machining of SiC_f_/SiC ceramic matrix composite test specimens was achieved, validating the feasibility of composite machining for SiC_f_/SiC materials. The main conclusions are as follows:

(1) The hybrid process effectively overcomes damage issues such as cracks and chipping associated with single-process grinding, as well as heat-affected zones and recast layers resulting from single-process laser processing. It significantly removes surface oxides generated by water-jet-guided processing, making the composition of the final machined surface closer to that of the substrate material. This demonstrates that the hybrid process enables efficient, low-damage machining of SiC_f_/SiC ceramic matrix composite test specimens.

(2) The water-jet-guided laser–ultrasonic grinding hybrid process combines the high material removal efficiency of water-jet-guided laser with the precision finishing advantages of ultrasonic grinding, outperforming single-process methods in both machining quality and efficiency. Employing 33 W laser power for water-jet-guided laser roughing and ultrasonic precision grinding at a feed rate of 500 mm/min achieves optimal overall machining quality while ensuring high efficiency.

(3) The hole diameter deviation (<0.017 mm), wall surface roughness (Sa < 1.815 μm), and machining efficiency (5.25 min/hole) achieved through the composite process outperform those of either ultrasonic grinding or water-jet-guided laser processing alone.

(4) Composite machining processes, involving secondary clamping, require consideration of coordinate reference alignment and secondary clamping issues. They are suitable for machining high-quality holes and hole clusters. When formulating actual process plans, specific analysis must be conducted based on different process objectives and machining characteristics. Integrating water-jet-guided processing with ultrasonic grinding achieves a comprehensive effect of high quality, high efficiency, and low cost.

## Figures and Tables

**Table 1:** Water-jet-guided laser drilling process parameters.

Serial Number	Power (W)	Feed Rate (mm/min)
1	20	200
2	25	
3	30	
4	33	

**Table 2:** Ultrasonic-assisted grinding process parameters for helical hole machining.

Serial Number	Feed Rate (mm/min)	Radial Cutting Depth (mm)
1	50	1.4
	50	0.1
2	200	1.4
	200	0.1
3	500	1.4
	500	0.1
4	500	1.4
	200	0.1

**Table 3:** Hole-making parameters of water-jet-guided laser precision grinding composite process.

Water-Jet Guided Laser Drilling, ⌀2.8 mm			Ultrasonic Grinding Hole Diameter ⌀3 mm	
Serial Number	Power (W)	Feed Rate (mm/min)	Feed Rate (mm/min)	Radial Cutting Depth (mm)
1	25	200	50,500	0.1
2	30			
3	33			

**Table 4:** Surface roughness value Sa of water-jet-guided processed hole walls.

Power (W)	Project	Surface Roughness (μm)		
		Position 1	Position 2	Position 3
25	Hole 1	2.004	2.013	2.046
	Hole 2	2.012	2.022	2.008
	Hole 3	2.008	2.003	2.016
	Average	2.015		
	Standard deviation	0.012		
30	Hole 1	2.609	2.582	2.589
	Hole 2	2.598	2.613	2.619
	Hole 3	2.599	2.618	2.628
	Average	2.606		
	Standard deviation	0.014		
33	Hole 1	2.695	2.732	2.686
	Hole 2	2.665	2.712	2.726
	Hole 3	2.678	2.716	2.738
	Average	2.705		
	Standard deviation	0.024		

**Table 5:** Water-jet-guided laser drilling aperture size.

Power (W)	Project	Inlet (mm)	Outlet (mm)
25	Hole 1	2.972	2.968
	Hole 2	2.978	2.962
	Hole 3	2.972	2.938
	Average	2.974	2.956
	Hole Diameters deviation	0.04	
	Standard deviation	0.012	
30	Hole 1	2.988	2.969
	Hole 2	2.976	2.965
	Hole 3	2.97	2.949
	Average	2.978	2.961
	Hole Diameters deviation	0.039	
	Standard deviation	0.011	
33	Hole 1	2.996	2.972
	Hole 2	2.982	2.965
	Hole 3	2.97	2.958
	Average	2.983	2.965
	Hole Diameters deviation	0.038	
	Standard deviation	0.012	

**Table 6:** Surface roughness value Sa of ultrasonic-milled hole walls.

Feed Rate (mm/min)	Project	Surface Roughness (μm)		
		Position 1	Position 2	Position 3
50	Hole 1	2.155	2.148	2.152
	Hole 2	2.159	2.146	2.155
	Hole 3	2.149	2.158	2.165
	average	2.154		
	Standard deviation	0.006		
200	Hole 1	2.4	2.426	2.398
	Hole 2	2.396	2.426	2.389
	Hole 3	2.432	2.469	2.418
	average	2.417		
	Standard deviation	0.023		
500	Hole 1	2.824	2.843	2.932
	Hole 2	2.802	2.936	2.964
	Hole 3	2.808	2.836	2.857
	average	2.867		
	Standard deviation	0.057		

**Table 7:** Ultrasonic grinding hole diameter.

Feed Rate (mm/min)	Project	Inlet (mm)	Outlet (mm)
50	Hole 1	2.984	2.965
	Hole 2	2.977	2.963
	Hole 3	2.961	2.962
	Average	2.974	2.963
	Hole Diameters Deviation	0.023	
	Standard deviation	0.008	
200	Hole 1	2.996	2.976
	Hole 2	2.982	2.965
	Hole 3	2.965	2.961
	Average	2.981	2.967
	Hole Diameters Deviation	0.035	
	Standard deviation	0.011	
500	Hole 1	3	2.989
	Hole 2	2.98	2.967
	Hole 3	2.958	2.95
	Average	2.979	2.969
	Hole Diameters Deviation	0.05	
	Standard deviation	0.015	

**Table 8:** Surface roughness value Sa of water-jet guidance–ultrasonic grinding of composite hole walls.

Processing Parameters	Project	Surface Roughness (μm)		
		Position 1	Position 2	Position 3
P = 33 W F = 50 mm/min	Hole 1	2.295	2.286	2.306
	Hole 2	2.316	2.296	2.288
	Hole 3	2.298	2.327	2.316
	Average	2.303		
	Standard deviation	0.013		
P = 33 W F = 500 mm/min	Hole 1	1.815	1.826	1.812
	Hole 2	1.902	1.798	1.826
	Hole 3	1.826	1.863	1.868
	Average	1.837		
	Standard deviation	0.031		

**Table 9:** Water-jet-guided laser–ultrasonic grinding composite hole-making diameter.

Feed Rate (mm/min)	Project	Inlet (mm)	Outlet (mm)
50	Hole 1	2.968	2.976
	Hole 2	2.96	2.972
	Hole 3	2.951	2.965
	Average	2.960	2.971
	Hole diameters deviation	0.025	
	Standard deviation	0.008	
500	Hole 1	2.963	2.968
	Hole 2	2.961	2.961
	Hole 3	2.953	2.951
	Average	2.959	2.960
	Hole diameters deviation	0.017	
	Standard deviation	0.005	

**Table 10:** Processing time statistics for water-jet-guided laser drilling at different power levels.

Serial Number	Power (W)	Feed Rate (mm/min)	Processing Time (min)
1	20	200	/
2	25		12
3	30		6
4	33		3

**Table 11:** Statistics on ultrasonic-assisted grinding spiral hole machining time.

Serial Number	Feed Rate (mm/min)	Radial Cutting Depth (mm)	Processing Time (min)
1	50	1.4	11
	50	0.1	22.5
2	200	1.4	2.8
	200	0.1	5.6
3	500	1.4	1.12
	500	0.1	2.25
4	500	1.4	1.12
	200	0.1	5.6

**Table 12:** Time statistics for water-jet-guided laser–ultrasonic composite hole formation.

Processing Steps	Process 1: Water-Guided Laser Drilling			Process 2: Ultrasonic-Assisted Grinding of Helical Holes			
Parameters	Power (W)	Feed Rate (mm/min)	Processing Time (min)	Feed Rate (mm/min)	Pitch (mm)	Machining Allowance (mm)	Processing Time (min)
1	33	200	3	500	0.01	0.2	2.25

**Table 13:** Comparison of processing method metrics.

Project	Water-Jet Guided Processing	Ultrasonic Grinding	Water-Jet Guided–Ultrasonic Grinding Hybrid Processing
Primary processing parameters	P = 33 W, F = 200 mm/min One-step machining to ⌀3.0 mm	F = 500 mm/min, first step machining to ⌀2.8 mm F = 200 mm/min, second step machining to ⌀3.0 mm	P = 33 W, first step machining to ⌀2.8 mm F = 200 mm/min, second step machining to ⌀3.0 mm
Surface roughness (μm)	2.705	2.417	1.837
Hole diameter deviation (mm)	0.04	0.05	0.017
Hole wall quality	Damage depth ≤ 90 μm	Damage depth ≤ 10 μm	No damage
Processing efficiency (min/hole)	3	6.72	5.25

## Data Availability

The original contributions presented in this study are included in the article material. Further inquiries can be directed to the corresponding author.

## References

[1] Ran Y., Kang R., Dong Z., Jin Z., Bao Y. (2023). Ultrasonic assisted grinding force model considering anisotropy of SiC_f_/SiC composites. Int. J. Mech. Sci..

[2] Chen J., An Q., Gong Q., Zeng D., Chen M. (2023). Machinability Improvement in Milling of SiC_f_/SiC Composites Based on Laser Controllable Ablation Pretreatment. J. Eur. Ceram. Soc..

[3] Sun D.R., Wang G., Li Y., Yu Y., Shen C., Wang Y., Lu Z. (2024). Laser drilling in silicon carbide and silicon carbide matrix composites. Opt. Laser Technol..

[4] Subasi L., Gokler M.I., Yaman U. (2023). A comprehensive study on water jet guided laser micro hole drilling of an aerospace alloy. Opt. Laser Technol..

[5] Zhang Y., Qiao H., Zhao J., Cao Z., Yu Y. (2020). Numerical simulation of water jet–guided laser micromachining of CFRP. Mater. Today Commun..

[6] Cheng B., Ding Y., Li Y., Yang L. (2022). Theoretical and Experimental Investigation on SiC/SiC Ceramic Matrix Composites Machining with Laser Water Jet. Appl. Sci..

[7] Cheng B., Ding Y., Li Y., Li J., Xu J., Li Q., Yang L. (2021). Coaxial helical gas assisted laser water jet machining of SiC/SiC ceramic matrix composites. J. Mater. Process. Technol..

[8] He J., Su H., Qian N., Xu P. (2022). Machining Performance Analysis of Rotary Ultrasonic-Assisted Drilling of SiC_f_/SiC Composites. Crystals.

[9] Huang B., Wang W.-H., Xiong Y.-F., Wu X.-F., Liu J.-T., Liu C., Wang D.-H. (2023). Investigation of force modeling in ultrasonic vibration-assisted drilling SiC/SiC ceramic matrix composites. J. Manuf. Process..

[10] Sun H., Dong Z., Yang F., Bao Y., Kang R., Sun J. (2026). Tool Wear Mechanism in Ultrasonic-Assisted Grinding of SiC_f_/SiC Composites. Ultrasonics.

[11] Gu C., Zhou Y., Hao X., Zhou M. (2026). A Study on the Grinding Forces and Inlet Defect Formation Mechanism in Ultrasonic Vibration Spiral Milling and Grinding Hole Making of SiC_f_/SiC Composite Materials. Int. J. Adv. Manuf. Technol..

[12] Dong Z., Zhang H., Kang R., Ran Y., Bao Y. (2022). Mechanical Modeling of Ultrasonic Vibration Helical Grinding of SiC_f_/SiC Composites. Int. J. Mech. Sci..

[13] Chen G., Wang J., Fu K., Chen J., Han Z., Ren L. (2025). Quality Enhancement and Defect Suppression in SiC/SiC Composites by Ultrasonic Vibration-Assisted Helical Grinding. Mater. Today Commun..

[14] Lin H., Zhou M., Wang H., Bai S. (2023). Investigation of Cutting Force and the Material Removal Mechanism in the Ultrasonic Vibration-Assisted Scratching of 2D-SiC_f_/SiC Composites. Micromachines.

[15] Huang B., Wang W., Jiang R., Xiong Y., Liu C. (2022). Experimental Study on Ultrasonic Vibration–Assisted Drilling Micro-Hole of SiC_f_/SiC Ceramic Matrix Composites. Int. J. Adv. Manuf. Technol..

[16] Wang C., Chen J., Zhang X., Wang T., Yang L., An Q., Ming W., Chen M. (2023). Effects of Ultrasonic Vibration Assisted Milling with Laser Ablation Pretreatment on Fatigue Performance and Machining Efficiency of SiC_f_/SiC Composites. J. Eur. Ceram. Soc..

[17] Kong X., Liu S., Hou N., Zhao M., Liu N., Wang M. (2022). Cutting Performance and Tool Wear in Laser-Assisted Grinding of SiC_f_/SiC Ceramic Matrix Composites. Mater. Res. Express.

[18] Hu T., Yuan S., Wei J., Zhou N., Zhang Z., Zhang J., Li X. (2024). Water Jet Guided Laser Grooving of SiC_f_/SiC Ceramic Matrix Composites. Opt. Laser Technol..

[19] Yin W., Yu Z., Xing G., Yang F., Dong Z. (2025). Study on the Microstructure Evolution and Ablation Mechanism of SiCp/Al Composites Processed by a Water-Jet Guided Laser. Materials.

[20] Cai X., Qin X., Niu J., Kang R., Dong Z., Bao Y., Ma G., Niu F. (2026). Effects of Laser Energy Uniformization via Static Beam Shaping on Ablation Behavior and Mechanism of SiC_f_/SiC Composites. J. Mater. Process. Technol..

[21] Song J., Wang B., Jiang Q., Hao X. (2025). Study on Laser Ablation Quality and Removal Mechanism of SiC_f_/SiC Composites by Wavelength and Thermal Effect Equation. Int. J. Appl. Ceram. Technol..

[22] (2021). Geometrical product specifications (GPS) — Surface texture: Areal—Part 2: Terms, definitions and surface texture parameters.

[23] Wei J., Yuan S., Yang S., Gao M., Fu Y., Hu T., Li X., Fan X., Zhang W. (2024). Waterjet-Guided Laser Processing of SiC/SiC Ceramic Matrix Composites to Obtain High Cleanliness and Low Oxidation Damage Characteristics Surfaces. Surf. Coat. Technol..

[24] Gao M., Yuan S., Wei J., Niu J., Zhang Z., Li X., Zhang J., Zhou N., Luo M. (2024). Optimization of Processing Parameters for Waterjet-Guided Laser Machining of SiC/SiC Composites. J. Intell. Manuf..

